# A single-model quality assessment method for poor quality protein structure

**DOI:** 10.1186/s12859-020-3499-5

**Published:** 2020-04-25

**Authors:** Jianquan Ouyang, Ningqiao Huang, Yunqi Jiang

**Affiliations:** 1 key Laboratory of Intelligent Computing & Information Processing, Ministry of Education, Xiangtan University, Xiangtan, China; 2 College of Chemistry, Xiangtan University, Xiangtan, China

**Keywords:** Protein structure ranking, Protein model quality assessment, Poor quality protein structural, Linear combination

## Abstract

**Background:**

Quality assessment of protein tertiary structure prediction models, in which structures of the best quality are selected from decoys, is a major challenge in protein structure prediction, and is crucial to determine a model’s utility and potential applications. Estimating the quality of a single model predicts the model’s quality based on the single model itself. In general, the Pearson correlation value of the quality assessment method increases in tandem with an increase in the quality of the model pool. However, there is no consensus regarding the best method to select a few good models from the poor quality model pool.

**Results:**

We introduce a novel single-model quality assessment method for poor quality models that uses simple linear combinations of six features. We perform weighted search and linear regression on a large dataset of models from the 12th Critical Assessment of Protein Structure Prediction (CASP12) and benchmark the results on CASP13 models. We demonstrate that our method achieves outstanding performance on poor quality models.

**Conclusions:**

According to results of poor protein structure assessment based on six features, contact prediction and relying on fewer prediction features can improve selection accuracy.

## Background

Proteins are large, important biological molecules. Direct prediction of a protein’s tertiary structure based on amino acid sequence is a challenging problem that has a significant impact on modern biology and medicine. The results of such predictions play key roles in understanding of protein function, design of proteins for new biological functions, and research and development of new drugs. With the completion of the Human Genome Project, more proteins’ amino acid sequences have been analysed by genome-sequencing technologies. Although the number of known protein amino acid sequences is increasing rapidly, the number of experimentally determined structures has lagged far behind the speed of amino acid analysis [1]. Meanwhile, scientific researchers have continued exploring and practicing. The main experimental methods are currently X-ray crystallography [2], NMR (Nuclear Magnetic Resonance), and Cryo-EM [3]. These existing methods often require much time and expensive resources, which prevents the speed of experimental protein structure determination from keeping up with the explosive growth in the number of available sequences [4].

One major challenge in structure prediction is selection of the best model from a pool of generated models. Protein structure prediction applications such as Rosetta [5–8] generate a large number of models of highly variable quality, but it is difficult to predict which is the closest to the native structure. There are currently two main types of model ranking. The first type is consensus methods, which calculate the model’s average similarity score against that of other models and usually assume that the pool’s higher-quality models have higher similarity with the other models in the pool. Consensus methods can usually achieve better performance, but experiments have shown that to realize this advantage, a large number of structural models of the same protein are required. The time complexity of consensus methods is O(n^2). If the number of structural models of the same kind of protein target is very large, the time and resources required for its calculation also become huge. In addition, these methods do not work when evaluating a single or few structural models of the same kind of protein target. The other type of ranking method is single-model quality assessment, which does not need to rely on other structural models. Currently, most single-model quality assessment methods use physics-based knowledge and evolutionary information from the sequences and can produce extracted output in the formats of features from other tools. For example, ProQ3 [9] extracts several features from sequence and energy terms produced by Rosetta in a format that can serve as input to a Support Vector Machine model. Another single-model quality assessment method, DeepQA [10], applies a belief network to 16 features. It relies on features from other tools, so when those tools have errors, increasing dependency on them does not improve accuracy. In summary, the existing single-model assessment methods use dozens of features obtained from other tools, and the more inaccurate features may cause the prediction results to be less credible. In addition, most model quality assessment methods and energy scoring functions perform well when model quality is better, but they often have bad performance on models of poor quality.

Therefore, we hope to explore a single-model quality assessment method that uses a few features as input to choose the best quality examples possible from the poor quality model set, which becomes the original motivation. In this paper, we propose a novel method for assessment of poor quality models that combines physics-based knowledge, tertiary structure properties, and physical properties derived from amino acid sequences. The total score is obtained by simple linear combinations rather than complex models such as machine learning. After performing a linear fit and weight random search on a large amount of data, ab initio models were generated using Rosetta and poor quality models from the 13th Critical Assessment of Protein Structure Prediction (CASP13) were used for benchmarking. Our method shows outstanding performance on low-quality models.

## Results

### Performance comparison on FM domains

Parts of proteins with known tertiary structures have been obtained by experimental methods. In most cases, homologous proteins have similar structures. Homology modelling is an effective method if a template with high homology similarity can be found [11]. However, not all targets have similar sequences in the Protein Data Bank, so the advantage of using a template is not applicable in all cases. In such situations, we usually use ab initio modelling methods, which are difficult to predict, and the quality of their results is not good without any constraints. Therefore, this type of model is our ideal evaluation target.

We evaluated our method on the CASP13 dataset and compared it with other open source methods and some features of our method. We divided evaluation of the FM domains dataset into two stages. The first stage was the generation of 1000 decoys for each target using Rosetta ab initio. This simulated our method’s model selection ability using real low quality prediction results.

We used the standard evaluation metrics: average per-target correlation and average per-target quality based on GDT-TS. In addition, to compare the present method’s performance with that of other methods, we selected the top 5 models for each method. Then, the top 1 model and the best of the top 5 models were evaluated separately (Table 1). In cases where the quality of the evaluation model was poor, all evaluation metrics were better under our method than the other investigated methods. Our model’s performance was significantly better than that of other methods, especially in terms of the correlation and Z-score sum of the top 1 model. In addition, we used two typical machine learning models using the same input feature for evaluation. Although machine learning models used the same features as input, the performance was still worse than linear combination. Table 1 shows that the effects of the two weighting methods (Random Search and Linear Regression) are very similar. Therefore, in future research, we will only use search weights as comparison results.

**Table 1:** Comparison results of various QA methods from Stage 1

QA methods	Corr. on stage 1	Top 1 GDT-TS on stage 1 / Z-score sum	Best model GDT-TS on stage 1 / Z-score sum
Ours (Random Search)	0.42	27.02/59.62	29.43/89.74
Ours (Linear Regression)	0.42	27.42/59.62	29.18/89.74
Ours (Random Forest)	0.32	22.66/14.27	26.65/58.09
Ours (Multilayer Perceptron)	0.37	25.24/45.25	29.15/85.34
DOPE	0.21	23.06/22.11	26.53/51.61
GOAP	0.21	23.48/23.79	27.09/63.87
ProQ4	0.22	23.45/22.58	27.38/61.14
ProQ3D	0.22	23.88/33.54	26.87/64.30
DeepQA	0.21	22.65/14.88	26.07/51.27

Z-score is calculated from the GDT-TS of the selected model and the GDT-TS of all models of the target. Sum the Z scores of each target to get the Z-score sum

To evaluate the prediction models produced by various methods, we used the low-quality models submitted by the CASP13 teams for Stage 2. Considering that the models submitted by some teams were not complete, some of the PDB files had amino acid residues missing. Therefore, the L value of the contact penalty term and the N value of the binary classification penalty term were determined on the basis of the amino acid residue sequences parsed from the PDB files. As shown in Table 2, the TM-score was also used as one of the evaluation metrics. In terms of the average Pearson correlation and the results for the top 1 model and top 5 models, our method still performs better than other methods.

**Table 2:** Performance of various QA methods measured by GDT-TS and TM-scores (CASP13 FM domains, poor quality dataset)

QA methods	Corr. TM-score on stage2	Corr. GDT-TS on stage 2	Top 1 TM-score on stage 2 / Z-score sum	Top 1 GDT-TS on stage 2 / Z-score sum	Best model TM-score on stage 2 / Z-score sum	Best model GDT-TS on stage 2 / Z-score sum
Ours	0.79	0.80	44.91/45.43	39.58/49.17	51.33/66.94	44.16/66.47
DOPE	0.48	0.48	40.05/29.04	34.67/31.73	44.27/42.28	38.32/44.79
GOAP	0.40	0.42	33.70/11.66	28.94/11.87	42.77/39.61	36.89/40.07
ProQ4	0.47	0.43	32.70/13.74	27.87/12.80	43.93/45.57	37.70/45.93
ProQ3D	0.61	0.62	42.26/41.89	36.52/45.08	48.94/61.72	42.39/64.74
DeepQA	0.55	0.55	34.23/13.92	29.33/16.21	47.05/55.47	40.22/56.76

Z-score is calculated from the GDT-TS of the selected model and the GDT-TS of all models of the target. Sum the Z scores of each target to get the Z-score sum

### Performance comparison on TBM-hard domains

Generally, target domains with good server predictions have close template homologs and are classified as TBM. To evaluate the accuracy of our method on TBM domains, we benchmarked our method’s performance on TBM-hard domains from CASP13 models in which GDT-TS is not greater than 50. As shown in Table 3, our method achieves good performance in terms of correlations with both GDT-TS and TM-scores. Although the average per-target quality of ProQ3D on the top 1 match was better than ours, the different quality distributions of the ProQ3D model under different domains give our method an advantage in terms of Z-score sum. Our method’s per-target average quality on the top 5 models based on TM-score was better than that of all other QA methods.

**Table 3:** Performance of various QA methods measured by GDT-TS and TM-scores (CASP13 TBM-hard domains, poor quality dataset)

QA methods	Corr. TM-scores on stage2	Corr. GDT-TS On stage 2	Top 1 TM-score on stage 2 / Z-score sum	Top 1 GDT-TS on stage 2 / Z-score sum	Best model TM-score on stage 2 / Z-score sum	Best model GDT-TS on stage 2 / Z-score sum
Ours	0.72	0.72	49.22/22.93	41.10/21.45	55.88/28.86	45.16/28.66
DOPE	0.42	0.41	47.31/13.09	38.25/15.03	53.25/21.36	43.05/22.84
GOAP	0.34	0.36	45.51/12.67	37.00/14.07	50.89/20.15	41.12/20.78
ProQ4	0.44	0.48	36.26/1.39	27.76/0.99	41.25/21.85	53.14/23.34
ProQ3D	0.68	0.68	52.92/20.78	41.27/20.22	56.02/24.51	43.90/24.01
DeepQA	0.46	0.47	39.76/0.61	29.35/0.14	47.77/11.68	31.16/11.64

Z-score is calculated from the GDT-TS of the selected model and the GDT-TS of all models of the target. Sum the Z scores of each target to get the Z-score sum

## Discussion

The predicted distance potential can also be used for model quality assessment, which improves prediction accuracy greatly [12]. The more accurate the distance prediction, the better the quality of the models and the more accurate model quality assessment using distance potential. However, not all distances can be accurately predicted, leading to incorrect distance prediction that is not conducive to correct modelling. Our method uses more mature contact predictions as a feature to evaluate model quality. Contact prediction can reduce distance prediction error by confining distance prediction within a certain range. Poor quality structural prediction models often have low distance prediction accuracy. It is not optimal to use inaccurate distance predictions in model selection. Thus, accurate distance prediction is not required for quality assessment of poor quality models, and fuzzy contact prediction is more appropriate.

To reduce complexity as much as possible, our method uses only six features as input. Many current quality assessment methods use deep learning. Because the prediction results of other tools are often used as feature inputs, the use of too many features may not improve accuracy. Moreover, most tools have bad prediction performance in terms of correlations with poor quality models (e.g. Table 1). ML models training using inaccurate feature input may not generate good results, so we use as few features and as simple of a model as possible to achieve the best results. Nevertheless, when the predictions of the tools that our method relies on have significant errors, the effect is still not satisfactory. In future work, to avoid inaccurate tool predictions, we will consider intercepting contact information from aligned homologous protein structures and rely on physical and chemical features that can be directly extracted from the protein structure as much as possible.

## Conclusion

In this paper, we have developed a single-model quality assessment method of protein tertiary structure prediction for poor quality models. It ranks models using a linear combination of six features. This method achieves good performance on CASP13 submission models and ab initio models generated by Rosetta. We believe that contact prediction is important information and that using it appropriately could further improve performance on poor quality models.

## Methods

### Input features

To reduce model complexity and improve accuracy on poor quality models, we selected six features as inputs for our method. These features statistically describe the potential, physio-chemical, and structural attributes of a protein model. DOPE (Discrete Optimized Protein Energy) [13], a representative statistical potential scoring method, obtains relevant statistics about atomic distances from known native protein structures. It also assists researchers with quality assessment, supported by probability theory. GOAP (Generalized Orientation-dependent All-atom Potential) [14] is another scoring method based on atomic distance and angle-related statistical knowledge, which can supplement DOPE.

Secondary structure information is essential to describe the physio-chemical properties of proteins. Although the number of possible conformations of a polypeptide chain is quite large, the conformational space would be vastly reduced if the secondary structure can be determined. At present, the best secondary structure prediction methods can reach accuracy rates of more than 80%. In this study, we use PSSpredv4 [15, 16] to predict secondary structure and use it as a benchmark to evaluate structure. Another important physical property of proteins is solvent accessibility. Here, we use SSPro4 [17, 18] to predict the solvent accessibility of the target protein and then compare the result with the estimated structure to impart a scoring penalty. Finally, accurate contact predictions can help us to limit the number of conformational searches at atomic distance. In recent years, with the development of deep learning, prediction of protein contact has become more accurate. As a representative protein contact prediction method, we use RaptorX [19–22] to calculate the penalty term for contact.

### Preprocessing

Table 4 is a summary table of all features. The quality assessment method consists of six parts, and the total score is obtained by weighting the sum of each part:

$$ {\displaystyle \begin{array}{c}E={\omega}_1{E}_{dope}+{\omega}_2{E}_{goap}+{\omega}_3{E}_{s\mathrm{s}\_h}+\\ {}{\omega}_4{E}_{s\mathrm{s}\_e}+{\omega}_5{E}_{sa}+{\omega}_6{E}_{contact}\#(1)\end{array}} $$

**Table 4:** Six features of our method

Feature Name	Descriptions
DOAP score	A statistical potential score for assessment and prediction of protein structures.
GOAP score	A generalized orientation-dependent, all-atom statistical potential score.
Secondary structure penalty score for helix	Calculate different ratios of predicted alpha-helix from amino acid sequence and a model parsed by DSSP.
Secondary structure penalty score for strand	Calculate different ratios of predicted beta-strand from amino acid sequence and a model parsed by DSSP.
Solvent accessibility penalty score	The difference of solvent accessibility prediction and model parsed by DSSP.
Contact penalty score	The percentage of contact prediction matching with the model structure.

The *E*_*ss*_ prefix indicates the penalty term of the secondary structure, and there is subdivision between the α-helix and β-strand penalty terms, indicated by *E*_*ss* _ *h*_ and *E*_*ss* _ *e*_. The solvent accessibility penalty term is *E*_*sa*_. We predict this term by ACCPro4, which returns a sequence result for each residue in binary classification format, indicating each residue’s solubility. With a solvent accessibility threshold of 25%, each residue is normalized to the maximum exposed surface area calculated using DSSP [23]. *E*_*contact*_ indicates the contact penalty. After the contact prediction results are sorted by confidence level, the top L residue pairs are taken, where L is the length of the amino acid sequence. We then find the corresponding residue pair in the structure and calculate the distance between the residues’ Cβ atoms (Cα for GLY). If the actual distance is no more than 8 Å, the position is consistent with the predicted structure.

DOPE and GOAP use the number of amino acid residues for normalization. In the above binary classification penalty items, the penalty score is:

$$ \frac{\sum_{i=i}^N\left({x}_i\bigoplus {p}_i\right)}{N}\#(2) $$

Here, *x*_*i*_ is the parsing result of each amino acid residue, and *p*_*i*_ is the corresponding prediction. The penalty term calculates the XOR value between the prediction of the corresponding amino acid residue position and the parsing result. Occasionally, if a feature cannot be calculated because of a tool failure, its value is set to 0.5.

### Dataset and weights

We collected CASP12 models from the CASP website http://predictioncenter.org/download_area/CASP12/predictions. We also downloaded the corresponding score table from the CASP website. The official CASP uses GDT-TS [24] (based on the LGA [25] package) as an important evaluation criterion. We extracted GDT-TS from the score table as the quality score. After excluding targets without the score table file, all remaining data models contain 75 whole chain targets, and the average target has 400 decoys. In total, we used 28,879 structural decoys covering a wide range of qualities to search weights.

**Fig. 1:**
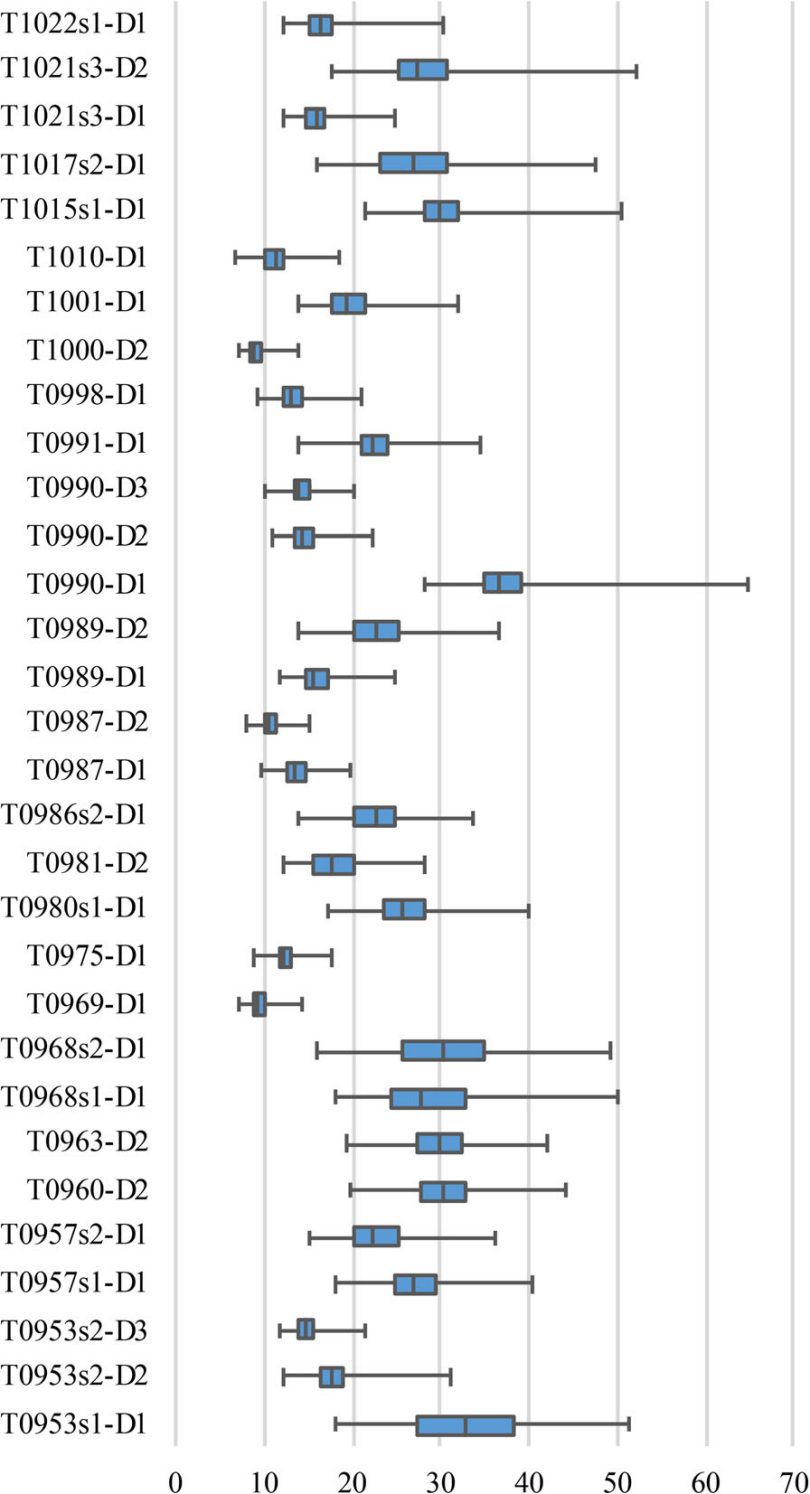
GDT-TS range of ab initio predictions made using Rosetta for targets in 31 FM (Free Modelling) domains of CASP13. The range of the generated decoys for each target is represented by a box plot, with the minimum, lower quartile, median, upper quartile, and maximum values in turn from left to right

To evaluate the performance of our method, we used 18,717 models in which GDT-TS was not greater than 50, they are from CASP13 human groups’ 31 FM (Free modelling) domains and 18 TBM (Template-based modelling)-hard domains (because of the limitation of RaptorX server on the length of the amino acid sequence, we discard T0985-D1). In addition, we used Rosetta ab initio to generate 1000 decoys for each FM domain from CASP13. For discontinuous domains, such as 77–134 and 348–520, we took the minimum and maximum of the interval to obtain 77–520. The GDT-TS score range for these data is shown in Fig. 1. Most of the generated decoys had GDT-TS scores below 50, and the lowest score was between 10 and 20. The best decoy quality in T0990-D1 was 64.8. Dataset can be downloaded from Google Drive, https://drive.google.com/file/d/12YscAvt1ZQCupdKopW8k9c1VUZu6NR03.

**Fig. 2:**
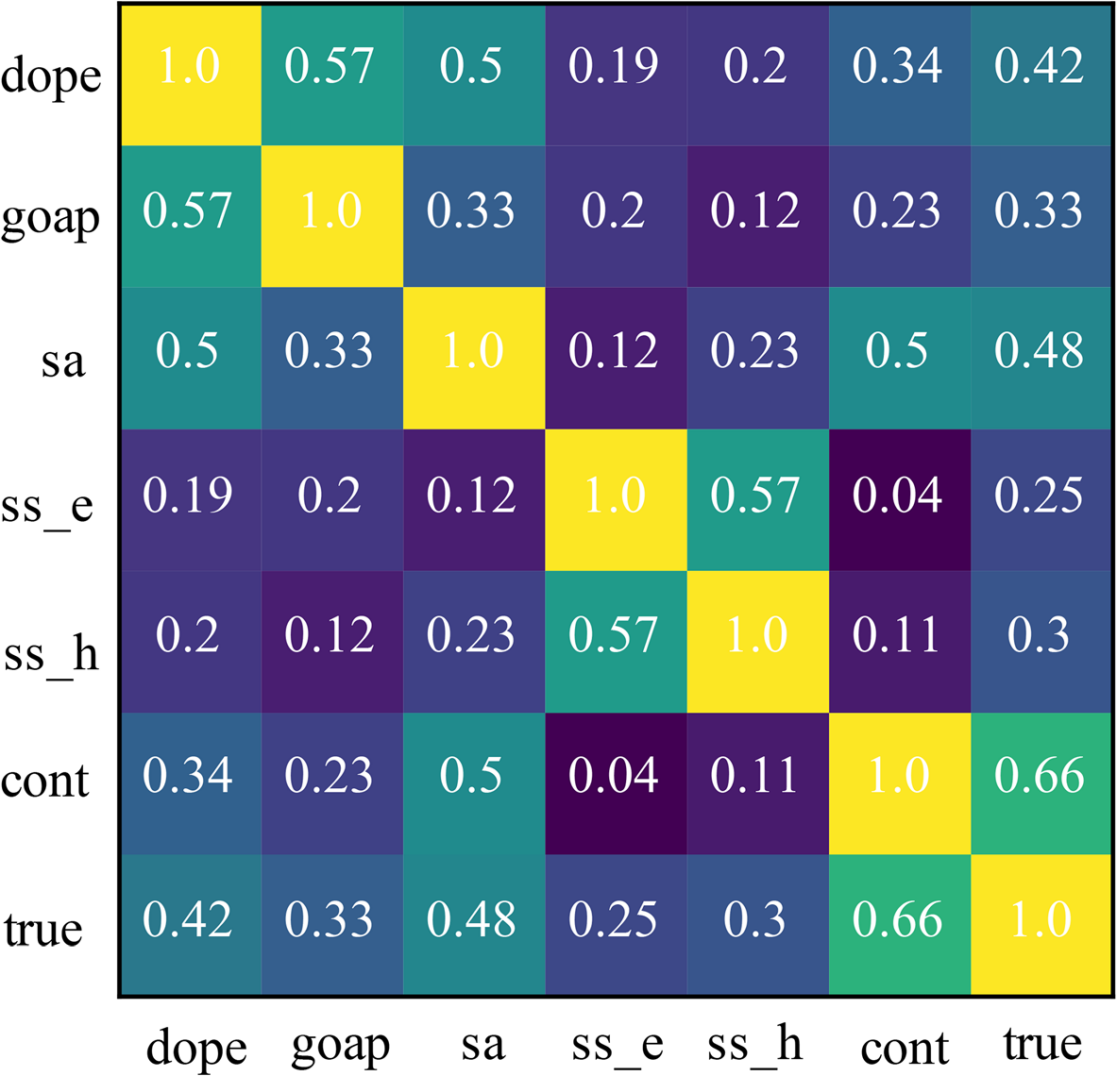
Correlation matrix of our method’s features obtained using CASP12 data, the true scores corresponding to the ground truth GDT-TS score

We developed two methods to obtain the weight values: one is to use linear regression, and the other is to perform a random search on the weights to obtain the maximum total correlation. We used 75 targets from the CASP12 dataset together to search for the optimized weights. The correlation matrix of each of our scores with the CASP12 data shows that the contact score had the best correlation, and the secondary structure folding score had the worst (Fig. 2). We calculated the MSE for GDT-TS using linear fit, obtaining values of 1.50, 1.79, 2.57, 2.54, 1.35, and 4.17 for DOPE, GOAP, SA, SS_E, SS_H, and Contact, respectively. Because our goal was to rank the structural models, we did not need the intercept value obtained from the linear regression. The goal of the weight random search is to optimize the sum of the correlation coefficients of all targets. Considering that the weights have a different impact on each target’s relevance, we used a random search method. The starting weights of the six features are float type variables in the range [0,1], and then random weight searches are performed to maximize the sum of the target correlation coefficients.

**Fig. 3:**
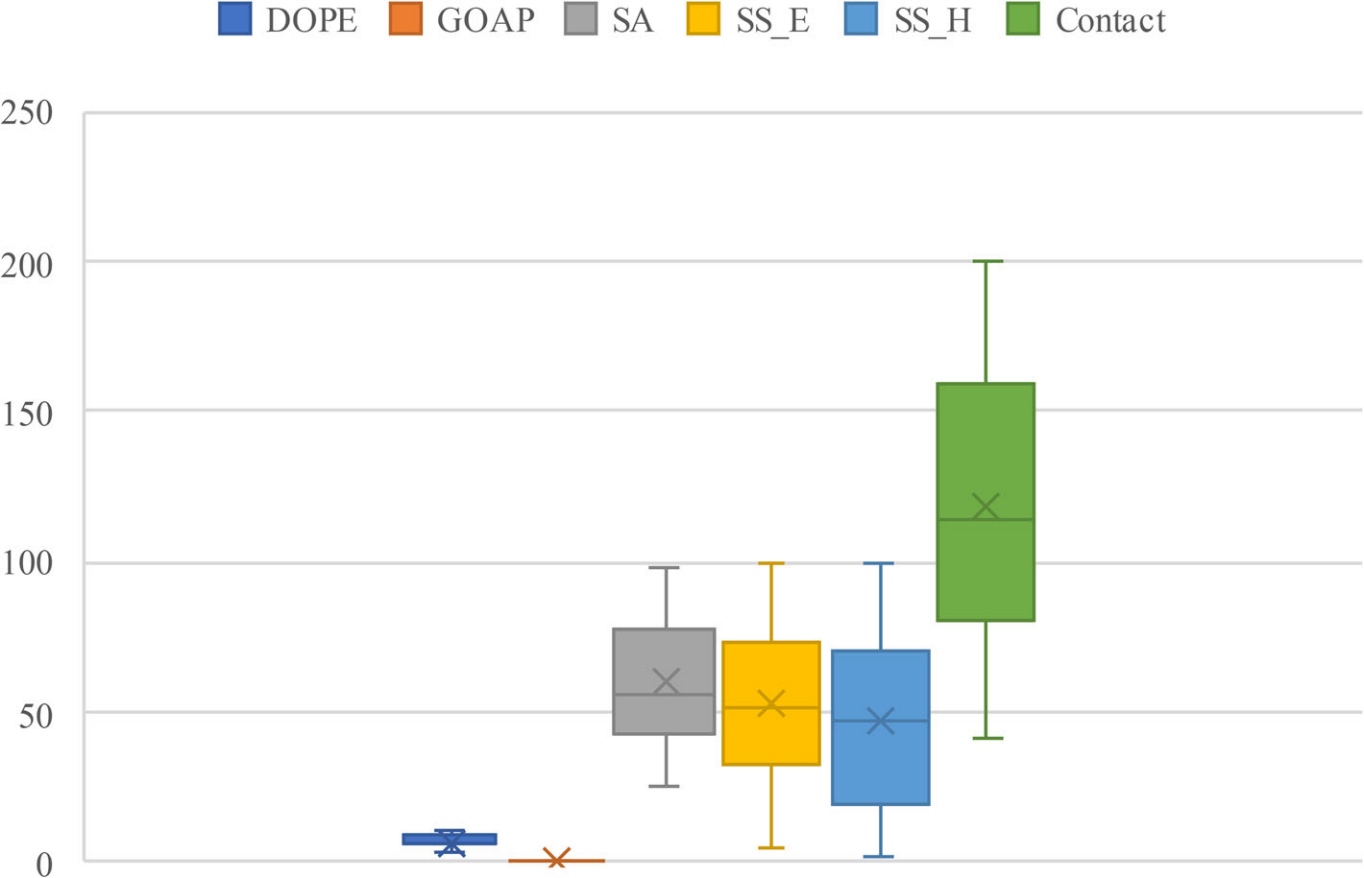
Ranges of the top 50 weight combinations obtained using CASP12 data and random search. The weight distribution range of each feature uses the box plot of the corresponding colour of the legend to represent the minimum, lower quartile, median, upper quartile, and maximum value

The random search was performed in 3 rounds, with each round lasting 10,000 steps. After each round of searching, we extracted the top 100 weight combinations and used their maximum and minimum values for each weight as the range for the next round of searching. If a weight’s search value is focused on the range boundary, the next round of search expands or reduces the weight’s range by 10 times. After three rounds of weight search, the sum of the correlation coefficients had risen to 54.39. We extracted the top 50 weight combinations in terms of correlation coefficients after the third round of searching (Fig. 3). The contact penalty term was given a larger weight, and the resulting distribution was more concentrated. A small weight was assigned to GOAP to reduce its impact on the results. We assigned the average value of each weight from the top 50 weight combinations as the final result. The final values were 4.96, 0.4, 70.65, 65.5, 76.5, and 168.16 for DOPE, GOAP, SA, SS_E, SS_H, and Contact, respectively.

## Data Availability

All data used in this study are available from the corresponding author upon request.
